# Editorial Notes

**Published:** 1895-05

**Authors:** 


### Editorial Notes.

Dr. Miller B. Hutchins has returned home after a ten days visit to New York.

Drs. Louis H. Jones, James L. Campbell and Alfred F. Harrington, of Atlanta, are in New York, taking special courses at the Post-Graduate and Polyclinic Hospital.

We regret to chronicle the death of Dr. A. G. Emory, of Acilla, Fla., which occurred on the 7th of May. The Record extends sympathy to the bereaved wife and family.

The National Association of Military Surgeons meets in Buffalo, N. Y., May 21st to 23d. The president is Dr. George N. Sternberg, Surgeon-General U. S. Army; the secretary, Dr. Eustathius Chancellor, 505 Olive street, St. Louis.

Dr. J. McFadden Gaston is in attendance at the meeting of the American Academy of Medicine in Baltimore, of which body he is president. Dr. Gaston will also remain in Baltimore during the meeting of the American Medical Association, and will be joined there by Drs. Dunbar Roy, J. M. Crawford, L. P. Stephens and Willis F. Westmoreland.

The following are the recently elected officers of the Medical Association of Georgia:

*President*—Dr. Frank M. Ridley, of LaGrange.
*First Vice-President*—Dr. W. H. Doughty, Jr., of Augusta.
*Second Vice-President*—Dr. M. L. Boyd, of Savannah.
*Secretary*—Dr. R. H. Taylor, of Griffin.
*Treasurer*—Dr. E. C. Goodrich, of Augusta.

